# Patient–provider communication quality: Socioeconomic disparities in smoking outcomes

**DOI:** 10.18332/tpc/184050

**Published:** 2024-03-11

**Authors:** Soumya Upadhyay, Jalen Jones

**Affiliations:** 1 Healthcare Administration and Policy, School of Public Health, University of Nevada Las Vegas, Las Vegas, Nevada, United States

**Keywords:** patient-provider communication, e-cigarettes, smoking

## Abstract

**INTRODUCTION:**

Patient–provider communication quality is instrumental for healthy outcomes in patients. The objective of this study is to examine the relationships between patient–provider communication quality and participant characteristics, perception of e-cigarette harmfulness, and smoking outcomes.

**METHODS:**

A pooled cross-sectional design was used on secondary data obtained from the Health Information National Trends Survey (HINTS) 5 from Cycle 1 through Cycle 4, from 2017–2022. Our final sample contained 3511 observations. Our outcome variable was the perception of electronic cigarette smoking status. The independent variable was patient–provider communication quality (PPCQ), measured from a series of questions with responses on a 4-item Likert scale (always, usually, sometimes, never). Demographic variables such as marital status, health insurance status, occupation status, and health-related variables were used as participant characteristics. Ordinal logistic regression models were used to examine the above relationships.

**RESULTS:**

Compared to males, females had lower odds of being in a higher category of perception of e-cigarette harmfulness compared to other categories of e-cigarette harmfulness (AOR=0.66; 95% CI: 0.57–0.76). Respondents who were non-Hispanic Black or Hispanic had lower odds of being in a higher category of perception of e-cigarettes compared to Whites (AOR=0.52; 95% CI: 0.49–0.78, and AOR=0.51; 95% CI: 0.41–0.65, respectively). Respondents who had higher education level compared to those with less than high school had lower odds (AOR=0.30; 95% CI: 0.17–0.51), and Hispanics compared to Whites had higher odds (AOR=1.59; 95% CI: 1.05–2.40), of being former smokers rather than current smokers.

**CONCLUSIONS:**

Providers should invest in staff training and development to target the populations that need conversations regarding e-cigarette usage.

## INTRODUCTION

E-cigarette use and perception of non-harmfulness have increased among youth, with 25% of high school students reporting past 30-day e-cigarette use in 2019^1^. The sales of e-cigarettes reached a peak of $436 million per 4-week period by August 2019^1^. These facts demonstrate the lack of perceived harmfulness of e-cigarettes among smokers. Quality patient–provider communication may increase the degree to which patients follow the recommendations of their healthcare providers and plays a vital role in encouraging healthy outcomes^2^. Quality patient–provider communication is defined as patient-centered communication – promoting a relationship in which patients are partners in the decision-making and management of their healthcare^3,4^. Studies have shown that patient–provider communication can positively influence patient outcomes, especially cancer which is expected to rise by 45% in the US by 2020 and is a serious outcome of smoking^5^. Quality patient–provider communication can include an exchange of opinions between patient and provider to help form the perception of patients about certain health-related habits, including perception of e-cigarettes^6^.

E-cigarettes are devices that vaporize nicotine to simulate smoking a combustible cigarette^7^. Awareness and use of e-cigarettes increased among youth in 2020, with 3.5 million US youth reporting past 30-day e-cigarette usage^8,9^. Research has shown that individuals who use e-cigarettes possess a greater understanding and awareness regarding the dangers associated with conventional cigarettes^10^. While smokers in the US have demonstrated strong support for regulations regarding safety quality, warning labels, and age restrictions, less restrictive e-cigarette policies were more likely to be supported by e-cigarette users who perceived them as less harmful than cigarettes^11,12^. These trends may indicate a shift from the use of traditional cigarettes to e-cigarettes and, in turn, an increased perception of less harmfulness compared to traditional cigarettes^13^. Providers may be able to mold patients’ perceived harmfulness of e-cigarettes through improved quality of communication^14^.

Quality patient–provider communication that is patient-centered may be determined by the patient’s perception of the quality of the interaction and exchange of information^15^. When compared to their White peers, providers are more likely to view racial and ethnic minorities as a group that does not follow directions, and providers may be more verbally dominant during patient–provider communication^16^. These biased situations and socioeconomic inequities may lead to health disparities, which may be related to racial and ethnic minorities reporting negative healthcare outcomes^17^. Additionally, it may influence the perception of e-cigarettes as a cessation tool among racial and ethnic minorities, as physicians have increased odds of recommending e-cigarettes if their patients ask about them first^18^. During lower quality instances of patient–provider communication, seen during interactions where the patient is a member of a racial or ethnic minority or has a lower education level, patients may feel less comfortable bringing up the topic of e-cigarettes and smoking in fear of being judged, limiting the physician’s influence on the patient’s perception of e-cigarette harmfulness^14^. The significance of the quality of patient–provider communication on health outcomes in general points to a need for a closer look into the relationship between patient–provider communication and smoking outcomes, as well as how sociodemographic factors play a role in this relationship.

Current literature has emphasized that the implementation of smoking cessation attempts is more manageable with the assistance of primary care providers. Providers who learn about the role of electronic cigarettes in reducing traditional smoking prevalence have improved communication with patients, especially with an increase in usage of other forms of nicotine delivery systems, such as electronic cigarettes^19-21^. While a few studies investigate electronic cigarette awareness, use, perceived harmfulness, and the associated socioeconomic disparities, there remains a gap in knowledge exploring patient–provider communication in association with perception of electronic cigarette harmfulness. The aim of this study is to examine the relationships between patient–provider communication quality and sociodemographic participant characteristics, perception of e-cigarette harmfulness, and smoking outcomes.

## METHODS

### Data and sample

A pooled cross-sectional design was used on secondary data obtained from Health Information National Trends Survey (HINTS) 5 from Cycle 1 through Cycle 4 and HINTS Cycle 6. The years of study ranged 2017–2022. HINTS is a nationally representative survey administered every two years by the National Cancer Institute (NCI) to adults aged ≥18 years, to monitor changes in health communication and information technology^22^. HINTS 5 Cycle 1 is a 2017 dataset with a total of 3191 complete responses (97.13% response rate). HINTS 5 Cycle 2 is a 2018 dataset updated in October 2020, in which the total completed responses were 3434 (98.0% response rate). HINTS 5 Cycle 3 is a 2019 dataset with 5247 complete responses (96.48% response rate), HINTS 5 Cycle 4 is a 2020 dataset with 3792 complete responses (98.11% response rate), and HINTS 5 Cycle 6 is a 2022 dataset with 6185 complete responses (98.92% response rate).

The sample of respondents included adults living in the US and were aged ≥18 years. After combining all responses from 2017 to 2022, we had a total of 13008 observations. After removing missing data, there were 3511 observations in the final dataset.

### Variables

*Dependent variables*

1. Perception of electronic cigarette harmfulness: was measured through the question in the survey that asked respondents to measure the perception of harmfulness at five levels on an ordinal scale (1=much more harmful, 2=more harmful, 3=just as harmful, 4=less harmful, 5=much less harmful).
2. Smoking status: was in the inclusion criteria, and a derived variable present in HINTS data based on self-reported smoking behavior questions – former smokers were classified as respondents who reported smoking at least 100 cigarettes in their lifetime but were not currently smoking; current smokers were classified as respondents who reported smoking at least 100 cigarettes in their lifetime and currently smoke on some days or daily.

*Independent variables*

Patient–provider communication quality (PPCQ) was measured from a series of questions with responses on a 4-item Likert scale (always, usually, sometimes, never). The questions focused on specific elements of communication with doctors, nurses, and other healthcare providers during the respondents’ visits within the past 12 months. The respondents were asked how often the provider: 1) gave them a chance to ask all the health questions they had; 2) gave the attention they needed to their feelings and emotions; 3) involved them in decisions about their healthcare as much as they wanted; 4) made sure they understood the things they needed to do to take care of their health; 5) explain things in a way they could understand; 6) spent enough time with them; and 7) helped them deal with feelings of uncertainty about their health or healthcare (https://hints.cancer.gov/data/survey-instruments.aspx). A construct was developed from this series of questions, similar to the perceived PPCQ (PPPCQ) variable in published literature^23^. Briefly, the Likert scale responses were recoded so that higher ratings corresponded to higher PPCQ, summed to create a composite score ranging 7–28, and rescaled by dividing the PPCQ composite score by 28. The PPCQ composite rescaled score ranged 0–1. The seven items demonstrated high internal consistency (Cronbach’s alpha=0.94), providing support for summation into a composite score. We examined the relationship between PPCQ and perception of e-cigarette harmfulness on the basis of gender (male, female), education level (less than high school, high school graduate, some college or post high school training, Bachelor’s degree or postgraduate), and race (Non-Hispanic White, Non-Hispanic Black, Hispanic, Non-Hispanic Asian, Non-Hispanic Other)^16^.

### Statistical analysis

HINTS 5 Cycle 1 through 5 and HINTS Cycle 6 data were merged in Stata using analytic recommendations downloadable with the HINTS dataset. The appended dataset was cleaned by removing missing values and negative values for factor variables. Variables were recoded based on our research questions. Descriptive analyses were used to describe all variables. Ordinal logistic regression analyses were conducted to analyze the relationships between the dependent and independent variables because our dependent variable perception of e-cigarette harmfulness is an ordinal variable with categories from 1 through 5. Adjusted odds ratios, which are antilog of the coefficients and reflect the adjustment by demographic characteristics of participants, were calculated. All statistical analyses were performed using Stata, version Stata 18^24^.

## RESULTS

Table 1 describes all the variables in the data. In terms of smoking status, approximately 71% were former smokers, and 29% were currently smoking. Respondents who perceived the harmfulness of electronic cigarettes as ‘just as harmful’ as cigarettes were in the highest category, at 58%. The lowest percentage of respondents were those who selected ‘much more harmful’ than cigarettes (about 4%). The independent variable, PPCQ composite, had a mean of 0.83 (SD=0.16). Among participant characteristics, there was a higher percentage of female respondents (60%) compared to males (40%). Most (51%) of the respondents belonged to the highest level of education (Bachelor’s degree or postgraduate). There was a higher percentage of Non-Hispanic White respondents (65%), compared to Non-Hispanic Black (13%) and Hispanic (14%). The highest percentage of respondents were married (51%), followed by divorced (15%). Most (97%) of the respondents had health insurance. About 55% of the respondents were employed, followed by 29% who were retired. About 73% of respondents perceived the quality of care provided to them to be either excellent or very good. The majority of the respondents seek care regularly from a provider (79%).

**Table 1:** Descriptive statistics of all variables in the dataset, 2017–2022 (N=3511)

*Variables*	*n*	*%*
**Dependent variables**		
**Smoking status**		
Former	3371	70.83
Current	1388	29.17
**Perceived harmfulness of electronic cigarettes**		
Much less harmful	850	10.73
Less harmful	780	9.85
Just as harmful	4565	57.64
More harmful	1438	18.16
Much more harmful	287	3.62
**Independent variables**		
**PPCQ composite rescaled,** mean (SD)	0.83	0.16
**Gender**		
Male	2352	39.54
Female	3596	60.46
**Education level**		
Less than high school	602	4.63
High school graduate	2019	15.52
Some college or post high school training	3734	28.71
Bachelor’s degree or postgraduate	6653	51.15
**Race**		
Non-Hispanic White	8508	65.41
Non-Hispanic Black	1688	12.98
Hispanic	1815	13.95
Non-Hispanic Asian	541	4.16
Non-Hispanic Other	456	3.51
**Marital status**		
Married	6631	50.98
Living as married	655	5.04
Divorced	2002	15.39
Widowed	1219	9.37
Separated	251	1.93
Single	2250	17.30
**Health insurance**		
Yes	7926	97.01
No	244	2.99
**Occupation**		
Employed	2019	55.16
Unemployed	102	2.79
Homemaker	177	4.84
Student	70	1.91
Retired	1063	29.04
Disabled	211	5.77
Other	18	0.49
**Quality of care**		
Excellent	4289	32.97
Very Good	5286	40.64
Good	2603	20.01
Fair	677	5.20
Poor	153	1.18
**Regular provider**		
Yes	6474	79.24
No	1696	20.76
**Age** (years), mean (SD)	55.29	16.74

Table 2 shows the results from ordinal logistic regression analysis between the relationship of perception of e-cigarette harmfulness and independent variables (Model 1). Compared to males, females had lower odds of being in a higher category of perception of e-cigarette harmfulness compared to other categories of e-cigarette harmfulness (AOR=0.66; 95% CI: 0.57–0.76). Respondents who had a Bachelor’s degree or higher had higher odds of being in a higher category of perception of e-cigarette harmfulness compared to those with less than high school education (AOR=1.61; 95% CI: 1.09–2.37). Respondents who were non-Hispanic Black and those who were Hispanic had lower odds of being in a higher category of perception of e-cigarettes compared to Whites (AOR=0.52; 95% CI: 00.49–0.78, and AOR=0.51; 95% CI: 0.41–0.65, respectively). Respondents who were married and those who were single had higher odds of being in a higher category of perception of e-cigarette harmfulness versus all the other combined categories of e-cigarette harmfulness (AOR=1.66; 95% CI: 1.13–2.44, and AOR=1.5; 95% CI: 1.23–1.83, respectively). Respondents who were older were significantly associated with being in a higher category of perception of e-cigarette harmfulness.

**Table 2:** Model 1: ordinal logistic regression analysis results for the association between perception of e-cigarette harmfulness and patient–provider communication quality, 2017–2022 (N=3511)

*Independent variables*	*AOR (95% CI)*	*p*
**Patient provider communication quality** (PPCQ)	0.58 (0.32–1.03)	0.065
**Gender**		
Male ®	1	
Female	0.66 (0.57–0.76)	<0.001
**Education level**		
Less than high school ®	1	
High school graduate	1.31 (0.87–1.96)	0.187
Some college or post high school training	1.29 (0.88–1.91)	0.187
Bachelor’s degree or postgraduate	1.61 (1.09–2.37)	<0.05
**Race and ethnicity**		
Non-Hispanic White ®	1	
Non-Hispanic Black	0.52 (0.49–0.78)	<0.001
Hispanic	0.51 (0.41–0.65)	<0.001
Non-Hispanic Asian	0.80 (0.56–1.14)	0.228
Non-Hispanic Other	0.99 (0.70–1.41)	0.990
**Marital status**		
Married ®	1	
Living as married	1.66 (1.13–2.44)	<0.05
Divorced	1.05 (0.86–1.28)	0.618
Widowed	0.86 (0.66–1.12)	0.284
Separated	1.04 (0.61–1.76)	0.881
Single	1.50 (1.23–1.83)	<0.001
**Health insurance**		
Yes ®	1	
No	1.11 (0.73–1.67)	0.613
**Occupation**		
Employed ®	1	
Unemployed	0.95 (0.61–1.46)	0.820
Homemaker	0.98 (0.70–1.37)	0.930
Student	1.11 (0.69–1.78)	0.644
Retired	1.01 (0.86–1.19)	0.882
Disabled	1.00 (0.72–1.39)	0.961
Other	0.87 (0.34–2.21)	0.780
**Perceived quality of care**		
Excellent ®	1	
Very good	0.98 (0.83–1.16)	0.865
Good	0.96 (0.75–1.23)	0.765
Fair	0.92 (0.61–1.39)	0.707
Poor	0.53 (0.22–1.29)	0.166
**Regular provider**		
Yes ®	1	
No	1.09 (0.91–0.09)	0.32
**Age** (years)	0.98 (0.97–0.99)	<0.001

AOR: adjusted odds ratio. ® Reference categories.

Table 3 shows the results from ordinal logistic regression analysis of the relationship between patient–provider communication quality and smoking status based on independent variables (Model 2). Respondents who had higher education level compared to those with less than high school had lower odds (AOR=0.30; 95% CI: 0.17–0.51), and Hispanics compared to Whites had higher odds (AOR=1.59; 95% CI: 1.05–2.40), of being former smokers rather than current smokers. Compared to married people, those who were divorced, separated, or single, had higher odds (AOR=1.52; 95% CI: 1.09–2.12) of being a former smoker rather than a current smoker. Retired people, compared to employed had lower odds (AOR=0.49; 95% CI: 0.36–0.66), and those who did not have a regular provider compared to those who had a regular provider, had higher odds (AOR=1.52; 95% CI: 1.11–2.09) of being a former smoker rather than a current smoker. Respondents who were older were significantly associated with being a former rather than a current smoker.

## DISCUSSION

The first aim of this study was to examine the relationship between patient–provider communication quality and perception of e-cigarette harmfulness. The second objective was to examine the relationship between patient–provider communication quality and smoking status. Multiple conclusions related to the independent variables can be drawn from this study.

**Table 3:** Model 2: ordinal logistic regression analysis results for the association between smoking status and patient–provider communication quality, 2017–2022 (N=3511)

*Independent variables*	*AOR (95% CI)*	*p*
**Patient–provider communication quality** (PPCQ)	0.77 (0.26–2.29)	0.644
**Gender**		
Male ®	1	
Female	0.10 (0.84–1.42)	0.484
**Education level**		
Less than high school ®	1	
High school graduate	0.71 (0.40–1.24)	0.238
Some college or post high school training	0.02 (0.36–1.05)	0.078
Bachelor’s degree or postgraduate	0.30 (0.17–0.51)	<0.001
**Race and ethnicity**		
Non-Hispanic White ®	1	
Non-Hispanic Black	1.40 (0.93–2.10)	0.104
Hispanic	1.59 (1.05–2.40)	<0.050
Non-Hispanic Asian	1.68 (0.73–3.86)	0.215
Non-Hispanic Other	1.01 (0.54–1.88)	0.970
**Marital status**		
Married ®	1	
Living as married	0.69 (0.36–1.35)	0.287
Divorced	1.52 (1.09–2.12)	<0.050
Widowed	0.68 (0.41–1.11)	0.138
Separated	2.58 (1.23–5.39)	<0.050
Single	2.20 (1.50–3.21)	<0.001
**Health insurance**		
Yes ®	1	
No	1.91 (0.98–3.81)	0.065
**Occupation**		
Employed ®	1	
Unemployed	1.87 (0.95–3.67)	0.068
Homemaker	0.67 (0.35–1.27)	0.221
Student	1.16 (0.30–4.48)	0.823
Retired	0.49 (0.36–0.66)	<0.001
Disabled	1.24 (0.80–1.93)	0.325
Other	2.04 (0.54–7.75)	0.292
**Perceived quality of care**		
Excellent ®	1	
Very good	1.09 (0.80–1.48)	0.588
Good	1.01 (0.64–1.58)	0.956
Fair	1.69 (0.81–3.51)	0.159
Poor	2.71 (0.67–10.81)	0.158
**Regular provider**		
Yes ®	1	
No	1.52 (1.11–2.09)	<0.001
**Age** (years)	0.97 (0.95–0.98)	<0.001

AOR: adjusted odds ratio. ® Reference categories.

### E-cigarette harmfulness perception

When compared to males, females are less likely to perceive e-cigarette harmfulness as more harmful than cigarettes. This might be because the dataset has a higher percentage of female respondents, about 20% more than male respondents. Another potential reason is that females, more often than males, may be more affected by advertisements and literature promoting the benefits of e-cigarette use. Females may be less likely to find e-cigarettes more harmful than cigarettes because e-cigarette use can be seen as a pathway to quit smoking, as conveyed by smoking cessation claims on e-cigarette advertising^25^.

The gender difference in perception of e-cigarette harmfulness may also be due to cigarette use being more likely to cause more harm to females than to males. For example, among women who smoke, the relative risk of lung cancer is significantly higher than among men who smoke^26^. This increased risk of disease continues to be disproportionate for females when compared to males in regard to chronic obstructive pulmonary disease (COPD) and cervical cancer^26^. The increased harmfulness of traditional smoking may be why e-cigarettes are less likely to be perceived as more harmful than cigarettes by women who are ever smokers.

When compared to those with less than a high school education, respondents who had higher education were more likely to perceive e-cigarettes as more harmful than cigarettes. This finding is in contrast to previous evidence that indicate that those with higher education were more likely to believe that e-cigarettes are less harmful than traditional cigarettes^27^. Our finding may be different than previous research because of the rapidly changing scope of e-cigarette use and perception, following the trend that perceived harmfulness of e-cigarette use is increasing alongside e-cigarette use^27^. This study population consists of only ever smokers, some of whom may have initiated smoking with e-cigarettes as they become more popular than traditional cigarettes. The FDA states that e-cigarettes are a safer alternative than cigarettes, so public health officials and governmental organizations may be lacking in their delivery of information regarding e-cigarettes as a ‘harm reduction’ method^28^.

Compared to their non-Hispanic White counterparts, respondents who were non-Hispanic Black were less likely to perceive e-cigarette harmfulness as more harmful than cigarettes. Whites were more likely to report smoking e-cigarettes than non-Hispanic Black adults or Hispanics, meaning that they are more likely to participate in e-cigarette smoking and experience harm from the use of e-cigarettes^29^. Multiple studies have shown that Whites, when compared to other races, are typically more aware of e-cigarettes in the first place^27^; therefore, other races, including non-Hispanic Blacks and Hispanics, may not have been as exposed to information regarding e-cigarette use. Massive public health measures have gone into conveying the extreme harms of cigarettes, including the Real Cost Campaign that, until recently, only focused on cigarette cessation^28^. Individuals unaware of e-cigarettes would be unlikely to consider them as more harmful than cigarettes, given the efforts to convey cigarettes as harmful. Additionally, the use of e-cigarettes involves a higher initial investment in the device itself^30^. However, over time, using e-cigarettes is cheaper than cigarettes^30^. Thus, non-Hispanic Black adults may prioritize the immediate cost-saving benefits of cigarettes over the long-term savings of e-cigarettes.

Compared to married respondents, those who are single are more likely to perceive e-cigarettes as more harmful than cigarettes. This finding leads to the fact that among single people, the prevalence of using any tobacco product is higher than among those who are living with a partner^31,32^.

Older participants are more likely to perceive e-cigarettes as much more harmful than cigarettes. This finding is likely due to older individuals having less exposure to e-cigarettes than cigarettes. Older participants may be weary of e-cigarette use due to being less familiar with the product. Additionally, as highlighted by the Real Truth Campaign, cigarette cessation advertising is often targeted toward youths^28^.

### Smoking status

The finding about those with higher education level being less likely to be a former smoker compared to those with lower education level, is probably because e-cigarette trends may be changing due to an increase in e-cigarette usage and the use of cigarettes being less popular recently. Also, the proportion of US citizens with higher education continues to grow, and academic stressors combined with the social nature of smoking on college campuses have increased^27^. Hispanic respondents were more likely to be former smokers compared to Whites, which was unexpected considering the long history of tobacco advertising targeted toward racial and ethnic minorities^33^. However, Whites are more likely to use e-cigarettes and cigarettes than Hispanics, signaling a potential cultural component to smoking status^29^.

Those who are divorced or single are less likely to be a former smoker than their married counterparts, which aligns with the prevalence of current smokers being higher among single adults than those who are married^31,32^. Married respondents typically engage in healthier lifestyle choices than those who are single/divorced. Students are less likely to be former smokers than those who are employed, which is likely due to the social aspect of smoking among university populations and academic-related stressors.

Respondents who do not have a regular provider, compared to those who do, are more likely to be former smokers. Although having a regular provider may improve clinical outcomes, the median age in our study was 55 years. Our results may be reflective of an older population who have smoked through their younger years and are already on the path to quitting, regardless of whether they have a regular provider or not. Nevertheless, quality patient–provider communication that includes physician advice in combination with screening can lead to healthier attitudes around smoking^34^. Older individuals are at an increased risk of smoking-related harms and other chronic conditions, incentivizing them to cease smoking.

### Limitations

Some limitations are worth noting. Our study sample contains data from 2017–2022. However, respondents in the first wave may not have been in the last wave, which leads to our inability to do a longitudinal study. This may lead to a lack of causal inferences.

In the HINTS dataset, information about the duration of time a patient has seen a provider is not available. Additionally, in this dataset, information about whether smoking cessation happens at the time patient–provider communication is taking place is also not available. Our assumption is that patient–provider communication has led to long-term perceptions of e-cigarette harmfulness, which would lead to smoking cessation at a certain point.

We also did not have information on how much time respondents have spent with their provider discussing e-cigarettes, the actual content of patient–provider communication, and whether the provider communicated positively or negatively about e-cigarettes to their patients. A primary data collection that includes the above factors beyond what is already included in our analyses would strengthen the analyses in future studies. Also, the trends surrounding demographics related to smoking prevalence, e-cigarette use versus traditional cigarette use, smoking awareness, etc., are changing and need further research.

## CONCLUSIONS

Findings from this study will inform healthcare providers to develop policies, training, and appropriate communication strategies surrounding e-cigarette use. This study encourages providers to ensure the necessary training of staff to stay up to date on e-cigarette literature as the impact of patient–provider communication on patient outcomes becomes more evident. Participants of certain demographic characteristics may need more targeted smoking-related conversations. As the smoking landscape continues to shift, organizations should foster greater investment in resources to improve the quality of patient–provider communication about the harmfulness of e-cigarettes.

## Data Availability

The data supporting this research are available from the authors on reasonable request.
